# Associations between Physical Fitness Index and Body Mass Index in Tibetan Children and Adolescents in Different High-Altitude Areas: Based on a Study in Tibet, China

**DOI:** 10.3390/ijerph191610155

**Published:** 2022-08-16

**Authors:** Yunjie Zhang, Fan Su, Yongjing Song, Jinkui Lu

**Affiliations:** 1 College of Education and Sports Sciences, Yangtze University, Jingzhou 434020, China; 2 College of Physical Education, China Three Gorges University, Yichang 443002, China; 3 School of Physical Education, Shangrao Normal University, Shangrao 334000, China

**Keywords:** high altitude, Tibetan, children and adolescents, body mass index, BMI, association analysis

## Abstract

Objective: To investigate the relationship between physical fitness index (PFI) and body mass index (BMI) of Tibetan children and adolescents in different high-altitude areas in Tibet, China. Methods: Using the stratified cluster sampling method, 3819 Tibetan children and adolescents from three different high-altitude areas including Nyingchi, Lhasa and Nagqu in the Tibet area of China were given grip strength, standing long jump, sitting forward bend, 50 m running and endurance running tests. One-way analysis of variance was used to compare the physical fitness index in different high-altitude areas. In addition, the method of curve regression analysis was used to analyze the relationship between PFI and BMI. Results: In general, the level of PFI in Nagqu, Tibet, China was lower than that in Nyingchi and Lhasa, and the levels of girls were generally lower than those of boys. The proportions of malnourished, normal, overweight and obese Tibetan boys in high-altitude areas were 11.8%, 79.7%, and 8.5%; those of girls were 3.3%, 82.3%, and 14.4%, respectively. The curve regression analysis showed that the model fitting of male Nyingchi, Lhasa, Nagqu and female Nyingchi, Lhasa, Nagqu were all significant (F values were 29.697, 34.709, 37.500, 9.123, 9.785, 6.939, *p* < 0.01). The relationship between BMI and PFI generally showed an inverted “U” curve relationship. Conclusion: The negative impact of overweight and obesity on physical fitness of Tibetan boys in high-altitude areas is significantly higher than that of girls, and the negative impact of overweight and obesity on physical fitness of boys in Lhasa and Nyingchi area is more significant than that in the Nagqu area. In the future, attention should be paid to Lhasa and the occurrence of overweight and obesity among Tibetan boys in Nyingchi area in order to prevent the sharp decline of physical fitness and promote the physical and mental development of Tibetan children and adolescents in high-altitude areas.

## 1. Introduction

The physical fitness development of children and adolescents is of great significance to the development of adolescence and the healthy development of adulthood in the future. Studies have confirmed that good physical fitness development of children and adolescents can effectively reduce the risk of various chronic cardiovascular diseases, hypertension, diabetes and all-cause mortality, and it can effectively prevent the occurrence of various chronic cardiovascular diseases [1,2]. A meta-analysis study showed that inverse dose–response associations of cardiorespiratory fitness(CRF) with all-cause, cardiovascular disease(CVD) and cancer mortality, as compared with lowest CRF, with highest CRF, the summary RRs for all-cause, CVD and cancer mortality were 0.47 (95 % CI 0.39 to 0.56), 0.49 (95% CI 0.42 to 0.56) and 0.57 (95% CI 0.46 to 0.70), respectively [1,2,3]. Studies have also confirmed that there is a close relationship between the physical fitness level of children and adolescents and the cognitive function, executive function and mental health of the brain. For example, a study of 422 Spanish children and adolescents confirmed that the higher the baseline level of cardiorespiratory fitness and muscular strength, which represents physical fitness, the better the working memory performance of children and adolescents (β range: 0.159 to 0.207, *p* < 0.012), and the inhibitory control was also better (β ranged from 0.168 to 0.263, *p* < 0.004), indicating that there was an independent correlation between the physical fitness level of children and adolescents with better working memory and inhibitory control, and it was suggested to encourage children and adolescents to actively improve their physical fitness level [4].

The physical fitness of children and adolescents mainly includes cardiorespiratory fitness, muscle strength, speed quality and flexibility. In the past, studies on the physical fitness of children and adolescents often used a certain physical fitness project for research, which could not fully reflect the physical fitness level of children and adolescents, so there were certain limitations [5,6,7]. For example, a study analyzed the relationship between cardiorespiratory fitness and waist circumference in Chinese children and adolescents; cardiorespiratory fitness was measured by 20 m shuttle run test, and the analysis showed that the relationship between cardiorespiratory fitness and waist circumference showed an inverted “U” curve [8]. With the deepening of research, in recent years, the use of physical fitness index (PFI), which comprehensively reflects the level of physical fitness, has gradually increased, and the relationship between physical fitness index and health has been deeply analyzed [9,10,11,12,13].

Research shows that in the past few decades, the physical fitness level of Chinese children and adolescents has generally shown a wave trend of “high-low-rebound”, but the overall level is still at a low level [14]. The results of the National Student Physical Health Survey in 2019 show that the physical fitness of Chinese children and adolescents generally shows a trend of improvement, but the problem of overweight and obesity is still serious and affects the development of physical fitness, which deserves attention [15]. Studies have shown that differences in the body size of children and adolescents have an impact on physical fitness [16,17,18]. For example, a study of Chinese and Japanese children and adolescents showed that the VO_2max_ level of Chinese children and adolescents was lower than that of Japanese children and adolescents, and the main reason was that Chinese children and adolescents were overweight (for boys: Chinese, 19.0%; Japanese, 11.0%, respectively. For girls: Chinese, 13.7%; Japanese, 8.5%, respectively) and there was a high obesity rate (for boys: Chinese, 13.1%; Japanese, 4.3%, respectively. For girls: Chinese, 5.5%; Japanese, 1.2%, respectively); these factors were closely related [19].

Tibetans mainly live in the Qinghai–Tibet Plateau, which is known as the “Roof of the World”, with an average altitude of more than 4000 m. Due to extreme environments such as plateau hypoxia, strong radiation, and large temperature differences, Tibetan children and adolescents in high-altitude areas have formed a special physique feature [20]. For example, studies have shown that Tibetan children and adolescents in high-altitude areas generally have lower levels of muscle strength than those in the plains [21]. In addition, some studies have confirmed that in order to better adapt to the high-altitude hypoxic environment, Tibetans in high-altitude areas have formed a wide and deep thoracic structure for a long time to adapt to the high-altitude hypoxic environment [22]. However, with the development of the economy and the continuous improvement of living standards, the problem of overweight and obesity among Tibetan children and adolescents has become more and more prominent, which has an impact on physical health [23].

In view of the special geographical environment of high-altitude areas, and the fact that previous studies have not found any research on the relationship between body mass index (BMI) and physical fitness status of Tibetan children and adolescents in different high-altitude areas, whether the relationship changes with altitude is unclear. To this end, this study conducted a PFI and BMI study on 3819 Tibetan children and adolescents in three different high-altitude areas, including Nyingchi (average elevation 3000 m), Lhasa (average elevation 3600 m), and Nagqu (average elevation 4100 m) in Tibet, China, analyzing the relationship between the two. In doing so, this study provides a targeted reference and basis for physical fitness promotion and exercise intervention of Tibetan children and adolescents in different high-altitude areas.

## 2. Materials and Methods

### 2.1. Participants and Procedures

From October to November 2021, the test and investigation of the subjects will be conducted in the Tibet region of China. The specific sampling method is divided into the following steps: First, according to the altitude, three different high-altitude areas, such as Nyingchi (average altitude 3000 m), Lhasa (average altitude 3600 m), and Nagqu (average altitude 4100 m), were selected as sampling test points. Second, 2 junior high schools and 2 high schools were randomly selected in each district as the schools for the test and survey. Third, in the selected schools, 3 teaching classes were randomly selected from each grade in each grade, and the local Tibetan students who lived in the plateau were selected as the research objects.

The specific inclusion criteria of the subjects are: (1) both parents of the subjects are Tibetans living in the plateau; (2) the subjects were born in Tibet and live in Tibet, China; (3) the subjects have no physical disease or psychological problems and are able to complete the test of this research; (4) they voluntarily accept the investigation of this research.

**Figure 1:**
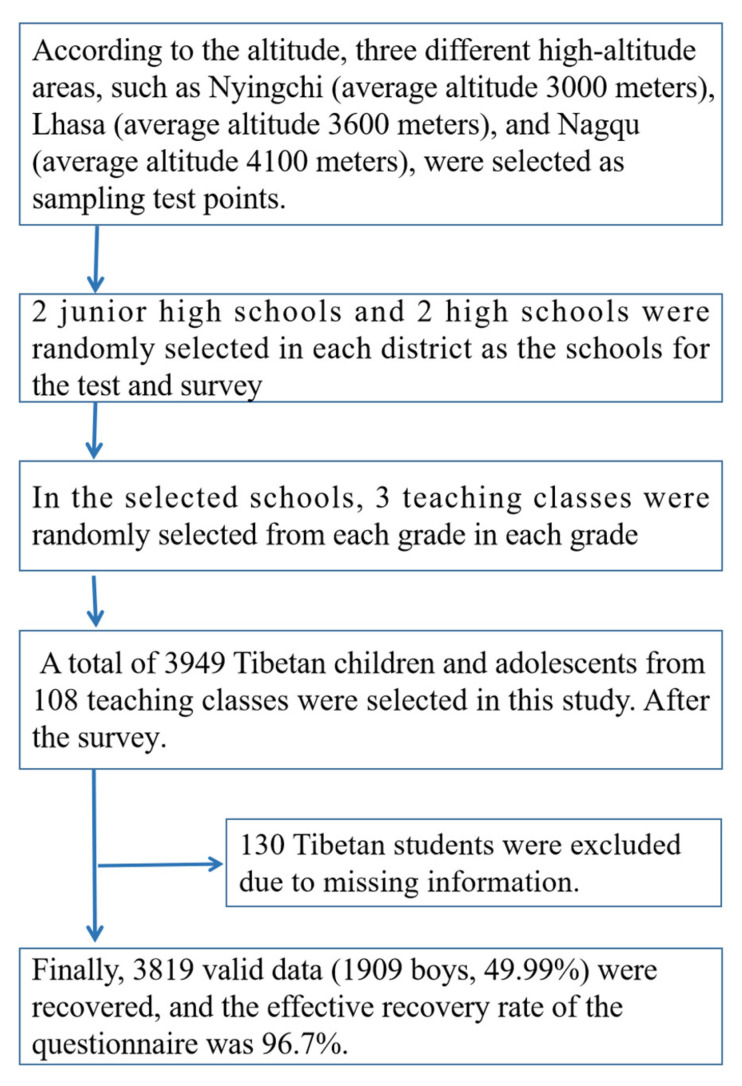
Sampling process of Tibetan children and adolescents in high altitude areas of China.

A total of 3949 Tibetan children and adolescents from 108 teaching classes were selected in this study. After the survey, 3819 valid data (1909 boys, 49.99%) were recovered, and the effective recovery rate of the questionnaire was 96.7%. The average age of the investigators was (15.51 ± 1.69) years for boys and (15.54 ± 1.69) years for girls. The sampling process of the specific subjects in this study is shown in Figure 1.

This research investigation was approved by the Human Ethics Committee of Changjiang University (202106784). Written informed consents from parents and students were obtained before the investigation. The questionnaire was filled out with an anonymous number to strictly protect the privacy of students.

### 2.2. Body Mass Index (BMI)

According to the height and weight of the test, the BMI is calculated, and the calculation formula is weight (Kg)/height (m)^2^. According to the BMI value and referring to the malnutrition screening of Chinese school-age children and adolescents (WS/T456-2014) [24] and the screening criteria for overweight and obesity in school-aged children and adolescents (WS/T586-2018) [25], which are divided into malnutrition, normal, overweight and obese. Therefore, overweight and obesity were combined into the overweight and obesity group for analysis. The specific value of the screening standard is divided according to different genders and different age groups. The classification standard is formulated by the Chinese government according to the actual situation of Chinese children and adolescents, and it is widely used [26,27].

### 2.3. Physical Fitness Index (PFI) 

The physical fitness test in this study includes grip strength, standing long jump, sitting forward bend, 50 m running, endurance running (1000 m for boys and 800 m for girls) and other physical fitness items. After the test, according to the mean and standard deviation of each item of the national male and female students aged 13–18 in the Chinese Student Physical Health Survey, the calculation of the Z-score for gender and age was carried out [28]. The specific calculation formula for the Z-score of each physical fitness item is (test value − National average)/national standard deviation, because the shorter the time for the 50 m run, the 1000 m run for girls and the 800 m run for girls, the better the performance. Therefore, the PFI is the Z value of grip strength + Z value of standing long jump + Z value of sitting forward flexion Value − Z value for 50 m run − Z value for endurance running.

### 2.4. Quality Control 

The items tested in this study include height, weight, grip strength, standing long jump, sitting forward bend, 50 m running, and endurance running (1000 m for boys and 800 m for girls). The tests are all conducted by trained physical education teachers, and each item is tested by a fixed staff. The testing instruments and methods are carried out in accordance with the requirements of the National Student Physical Health Survey and related research methods [29,30]. The test instrument is tested daily after calibration. The height is accurate to 0.1 cm, the weight and grip strength are accurate to 0.1 kg, the standing long jump and seated forward bend are 0.1 cm, the 50 m run is 0.1 s, and the 1000 m or 800 m run is 1 s.

### 2.5. Statistical Analysis 

The basic situation of the PFI of Tibetan children and adolescents in different gender and age groups in high-altitude areas is expressed as median, P25, and P75 digits. The PFI of Tibetan children and adolescents with different nutritional status is also expressed by median, P25, and P75 digits. Due to the non-normal distribution of PFI, the Kruskal–Wallis H test was used for comparison between different high-altitude regions (Nyingchi, Lhasa, Nagqu) and different body types. According to H value and P value, we can understand the differences in PFI among Tibetan children and adolescents in different high-altitude areas (Nyingchi, Lhasa, Nagqu) and different nutritional status (malnutrition, normal, overweight and obesity).

The relationship between BMI and PFI in boys and girls in different high-altitude areas (Nyingchi, Lhasa, Nagqu) was analyzed by curve regression analysis, with PFI as the dependent variable. BMI and the square of BMI were the independent variables, and the curve regression model was established. PFI = aBMI^2^ + bBMI + c is obtained, where a, b, and c are constants. Finally, our study derived a curve regression model of BMI and PFI for boys and girls by region at different high altitudes (Nyingchi, Lhasa, Nagqu), respectively.

The data were entered using EpiData3.0, the data after the entry were verified again by another group of students, and the data were imported into SPSS software (version 25.0; IBM Inc., Armonk, NY, USA) for processing and analysis. GraphPad Prism 8.0.2 was used (GraphPad Software, Inc., San Diego, CA, USA) for image creation. *p* < 0.05 was used as the two-sided test level.

## 3. Results

As can be seen in Table 1, in different high-altitude areas between the ages of 13 and 18, the overall median range of PFI in Nagqu is ((−5.17)–0.43), which are lower than those in Nyingchi ((−1.26)–0.65) and Lhasa ((−1.08)–1.21). In terms of gender, girls ((−1.26)–0.57) are generally lower than boys ((−5.17)–0.94). Except for the 17-year-old age group, there was no significant difference in PFI in different regions, but there were significant differences in other regions (H values 96.423, 11.397, 23.439, 22.260, 32.916, all *p* values < 0.01). Girls, except for the 15-year-old age group, had significant difference in PFI in different regions, while other significant differences were found (H values 8.679, 10.120, 11.465, 16.482, 7.690, all *p* values < 0.05).

Table 2 shows that in general, the proportions of malnutrition, normal, overweight and obese Tibetan boys in high-altitude areas are 11.8%, 79.7%, and 8.5%; the proportions for girls are 3.3%, 82.3%, and 14.4%, respectively. Among Tibetan children and adolescents aged 13–18, there were significant differences in the PFI of boys except for the 16- and 18-year-old age groups with different nutritional status (malnutrition, normal, overweight and obesity) (H values were 12.712, 36.462, 18.654, 10.833, all *p* values were <0.01). There was a significant difference in PFI among girls with different nutritional status only in the 13-year-old age group (H value 10.836, *p* < 0.01), and there was no significant difference in other age groups.

According to the change trend of PFI in different body types, it can be seen that the two generally show a curvilinear relationship. After stratifying according to different genders and altitudes, using PFI as the dependent variable and BMI and BMI^2^ as the independent variables, the curve regression analysis was carried out. The model fitting of Nyingchi, Lhasa and Nagqu with girls are all significant (F values are 29.697, 34.709, 37.500, 9.123, 9.785, 6.939, *p* values are all < 0.01) (Table 3).

The specific fitting model is:

Boys:

Nyingchi:PFI = −0.042BMI^2^ + 1.886BMI − 20.577 (*R*^2^ = 0.081);

Lhasa:PFI = −0.065BMI^2^ + 2.788BMI − 29.267 (*R*^2^ = 0.089);

Nagqu:PFI = −0.024BMI^2^ + 1.453BMI − 20.837 (*R*^2^ = 0.114);

Girls:

Nyingchi:PFI = −0.027BMI^2^ + 1.143BMI − 12.403 (*R*^2^ = 0.024);

Lhasa:PFI = −0.035BMI^2^ + 1.528BMI − 16.365 (*R*^2^ = 0.025);

Nagqu:PFI = −0.009BMI^2^ + 0.523BMI − 7.601 (*R*^2^ = 0.021).

**Figure 2:**
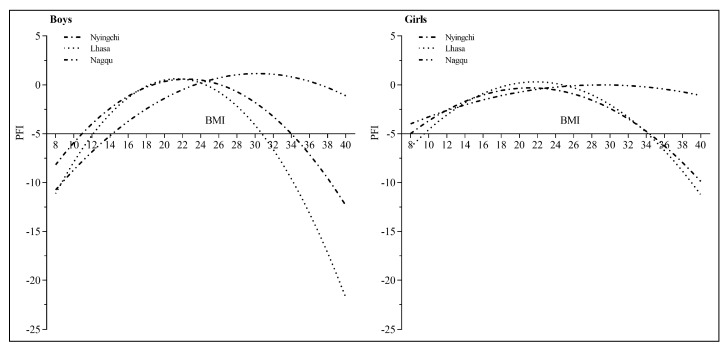
The relationship between body mass index and PFI of Tibetan children and adolescents of different genders at different altitudes.

According to the original data of BMI, the BMI of the test data is the largest change range of 7.96 kg/m^2^~39.75 kg/m^2^, so the equation-fitting curve with BMI as the abscissa (8–40 kg/m^2^) and PFI as the ordinate is shown in Figure 2. It can be seen that the relationship between BMI and PFI generally presents an inverted “U” curve relationship, and PFI is lower when BMI is lower or higher. Regional Tibetan children and adolescents have a more significant impact on physical fitness. Compared with girls, boys were more significant, especially in the Lhasa and Nyingchi regions, the effect of BMI on PFI was more obvious.

## 4. Discussion

The results of this study show that the overall PFI of Tibetan middle school girls in high-altitude areas is lower than that of boys, indicating that girls’ physical fitness performance is generally lower than that of boys, which is consistent with the conclusions of many studies [31]. The main reason for this result is related to gender. Studies have shown that boys are naturally active and spend more time participating in physical exercise than girls, resulting in higher levels of physical activity and higher PFI levels in boys than girls [32,33]. On the other hand, physical fitness is mainly composed of children and adolescents’ muscle strength and cardiorespiratory fitness, and boys’ muscle strength is higher than that of girls. Therefore, physical fitness has significantly higher performance in standing long jump, 50 m running, and endurance running. In girls, the PFI index reflecting the comprehensive level of physical fitness is higher for boys than girls, which is consistent with the conclusion of relevant research [34].

In different high-altitude areas, the PFI level of children and adolescents in Nagqu was the lowest. The reason for this result is that Nagqu is located at a high altitude of more than 4000 m, and the air hypoxia is more serious, which cannot guarantee the body’s demand for oxygen in the physical fitness test, resulting in low scores [35]. In addition, high-altitude radiation is strong, and students spend less time participating in outdoor exercise, which is also an important reason for the low level of physical fitness. Third, because students in Nagqu are often boarding students, students mainly eat on campus and consume a lot of snacks, which leads to an unsatisfactory nutritional status and is also an important reason for low physical fitness. On the contrary, the economic development level of the Lhasa and Nagqu regions is relatively good, the number of day students is relatively small, and their lives are more concerned by their parents, so their nutrition is more balanced, which leads to better physical fitness.

In terms of different nutritional status, in general, the PFI of malnutrition Tibetan children and adolescents in high-altitude areas is lower than that of normal and overweight and obese children and adolescents. In terms of the degree of influence, boys are more significant than girls. For example, taking Lhasa as an example, when the BMI of boys is 8 kg/m^2^, the PFI is −11.123, while the PFI of girls is −6.381. When the BMI of boys was 40 kg/m^2^, the PFI was −21.747, while the PFI of girls was only −11.245. It can be seen that the effect of BMI on PFI is more significant in boys than in girls. This result shows that the overall level of physical fitness of malnourished Tibetan children and adolescents in high-altitude areas is relatively low. It is suggested that in the future, we should pay more attention to the improvement and improvement of the physical fitness of malnourished students, and at the same time, we should reduce the proportion of Tibetan children and adolescents who are malnourished in high-altitude areas to improve the level of physical fitness. The reason for the lower PFI of the malnourished students is that the malnourished students have lower body muscle content, smaller muscle cross-sectional area, and thinner muscle fibers. In the process of participating in physical fitness tests such as grip strength, standing long jump, and 50 m running, students with normal weight and overweight tend to perform poorly, resulting in lower PFI levels [36,37]. The study shows that the physical fitness level of Chinese malnourished children and adolescents is lower than that of normal and overweight and obese students, which is consistent with the conclusion of this study [38].

The curve regression analysis of BMI and PFI showed that there was an inverted “U” curve relationship between BMI and PFI in Tibetan children and adolescents in high-altitude areas. Our findings are consistent with the conclusions of several studies [9,14]. Compared with girls, the curve relationship of boys is more “steep”, which also shows that the impact of malnutrition and obesity on physical fitness of Tibetan boys is significantly higher than that of girls; especially the impact of BMI on PFI in boys in Lhasa and Nyingchi is more obvious, namely steeper. It shows that the effect of BMI on PFI is more obvious in higher altitude areas, and there are gender differences. The reason is that the PFI level of Tibetan children and adolescents in Nagqu region of China is the lowest, and its variation range is small. However, the level of PFI of Tibetan children and adolescents in Lhasa and Nyingchi areas is relatively high, and the fluctuations of their PFI are easily affected by factors of overweight or obesity. Because the proportion of malnutrition among Tibetan children and adolescents in Lhasa and Nyingchi is lower, they are more affected by overweight and obesity factors on PFI. It suggests that we should pay more attention to the improvement and promotion of the physical fitness level of malnutrition and obese Tibetan boys in high altitude areas in the future. It can also be seen from the curve diagram of this study that the BMI of Tibetan obese middle school boys exceeds the normal range; that is, obesity has a more significant impact on PFI. Therefore, more attention should be paid to prevent the occurrence of overweight and obesity in boys and reduce the proportion of occurrence in order to promote the improvement of physical fitness level. In addition, from the perspective of different altitude areas, the increase in overweight and obesity in Tibetan boys in Lhasa and Nyingchi has a more significant impact on physical fitness than in Nagqu. This also suggests that we should pay special attention to the occurrence of overweight and obesity among Tibetan boys in Lhasa and Nyingchi in the future to prevent the sharp decline of physical fitness.

In conclusion, according to the results of our analysis of the association between BMI and PFI of Tibetan children and adolescents in different high-altitude areas, we have reason to believe that there is an inverted “U” curve relationship between the BMI and PFI of Tibetan children and adolescents in different high-altitude areas. However, the degree of influence between BMI and PFI was inconsistent with altitude. Our findings suggest that in the future, different interventions or public health promotion policies should be adopted for Tibetan children and adolescents according to different altitudes. For example, the Nagqu region of Tibet, China should focus on improving nutritional status and reducing malnutrition. For Tibetan children and adolescents in Lhasa and Nyingchi, China, the focus should be on weight control and reducing the incidence of overweight or obesity. Physical fitness promotion measures and public health policy development should vary or vary by altitude.

## 5. Conclusions

Our study is the first to analyze the relationship between BMI and PFI in Tibetan children and adolescents at high altitudes in China. The results showed that the relationship between BMI and PFI of Tibetan children and adolescents showed an inverted “U” curve. The negative impact of overweight and obesity on the physical fitness of Tibetan boys was significantly higher than that of girls, and the negative impact of overweight and obesity on physical fitness of boys in Lhasa and Nyingchi was more significant than that in Nagqu. In the future, attention should be paid to the occurrence of overweight and obesity among Tibetan boys in Lhasa and Nyingchi to prevent the sharp decline of physical fitness.

## Figures and Tables

**Table 1:** Comparison of PFI status of Tibetan children and adolescents of different genders at different altitudes (Median (P25, P75)).

Age (yrs)	Boys			*H*-Value	*p*-Value	Girls			*H*-Value	*p*-Value
	Nyingchi	Lhasa	Nagqu			Nyingchi	Lhasa	Nagqu		
13	0.03 (−1.6, 1.97)	−0.06 (−2.55, 1.62)	−5.17 (−7.55, −2.77)	96.423	<0.01	−0.13 (−1.78, 1.22)	0.03 (−1.29, 1.68)	−1.17 (−3.52, 0.27)	8.679	<0.05
14	0.03 (−1.70, 1.65)	−0.40 (−2.80, 1.66)	−1.37 (−3.50, 0.08)	11.397	<0.01	−0.23 (−1.64, 1.01)	−0.02 (−1.46, 2.28)	−1.16 (−3.12, 0.39)	10.120	<0.01
15	−0.42 (−2.82, 1.40)	−0.58 (−2.22, 1.56)	−2.88 (−4.56, −1.00)	23.439	<0.01	−1.26 (−3.12, 0.73)	−1.08 (−3.30, 0.94)	−1.10 (−2.43, 0.48)	0.068	0.967
16	0.65 (−1.04, 1.75)	0.94 (−0.33, 2.38)	−1.38 (−3.07, 1.93)	22.260	<0.01	−0.03 (−2.05, 1.44)	−1.08 (−3.30, 0.94)	−0.97 (−3.53, 1.62)	11.465	<0.01
17	0.38 (−0.78, 2.12)	0.84 (−1.25, 2.00)	−0.66 (−2.67, 1.57)	3.418	0.181	−0.47 (−1.84, 1.45)	0.57 (−0.86, 2.32)	−0.64 (−2.86, 0.40)	16.482	<0.01
18	0.56 (−0.80, 2.31)	1.21 (−0.21, 2.37)	−1.60 (−3.23, 0.65)	32.916	<0.01	−1.03 (−2.95, 1.19)	−0.48 (−1.89, 1.57)	0.43 (−0.83, 1.92)	7.690	<0.05

**Table 2:** Comparison of PFI of different nutritional status of Tibetan children and adolescents of different ages in high altitude areas (median (P25, P75)).

Age (yrs)	Boys			*H*-Value	*p*-Value	Girls			*H*-Value	*p*-Value
	Malnutrition	Normal	Overweight and Obesity			Malnutrition	Normal	Overweight and Obesity		
13	−3.31 (−5.62, −0.70)	−1.11 (−3.51, 1.48)	−1.02 (−5.12, 0.37)	12.712	<0.01	−3.02 (−4.85, −1.01)	−0.12 (−1.75, 1.4)	−0.55 (−2.53, 0.6)	10.836	<0.01
14	−3.38 (−4.61, −1.49)	−0.10 (−1.82, 1.63)	−1.10 (−3.73, 0.75)	36.462	<0.01	−1.71 (−3.52, −0.88)	−0.32 (−1.79, 1.18)	−0.43 (−2.02, 0.85)	3.815	0.148
15	−3.42 (−5.45, −1.20)	−0.64 (−2.78, 1.46)	−2.20 (−3.96, −1.12)	18.654	<0.01	−1.28 (−4.21, −0.43)	−1.07 (−2.94, 0.93)	−1.34 (−3.2, 0.44)	0.777	0.678
16	−1.65 (−4.47, 0.88)	0.61 (−1.53, 2.14)	0.04 (−2.03, 0.80)	3.874	0.144	1.41 (−0.44, 3.28)	−0.04 (−2.15, 2.10)	−0.44 (−1.99, 1.50)	0.223	0.894
17	−1.24 (−2.46, 1.07)	0.51 (−1.04, 2.10)	−0.07 (−2.81, 1.73)	10.833	<0.01	−0.37 (−2.12, 1.02)	0.04 (−1.54, 1.99)	−0.74 (−2.30, 0.55)	4.580	0.101
18	0.10 (−1.73, 1.32)	0.38 (−1.46, 2.09)	−0.77 (−2.15, 1.21)	1.634	0.442	−0.83 (−2.06, 1.88)	−0.18 (−2.06, 1.74)	−0.65 (−2.21, 1.68)	2.825	0.243

**Table 3:** Curve regression analysis of body mass index and PFI of Tibetan children and adolescents of different genders at different altitudes.

Gender	Area	Arguments/Constants	*B*-Value	Standard Error	*β*-Value	*t*-Value	*p*-Value
Boys (*n* = 1909)	Nyingchi	BMI	1.886	0.245	2.034	7.691	<0.01
		BMI^2^	−0.042	0.005	−2.032	−7.683	<0.01
		(Constants)	−20.577	2.726		−7.547	<0.01
	Lhasa	BMI	2.788	0.341	2.693	8.171	<0.01
		BMI^2^	−0.065	0.008	−2.736	−8.301	<0.01
		(Constants)	−29.267	3.690		−7.932	<0.01
	Nagqu	BMI	1.453	0.240	1.146	6.064	<0.01
		BMI^2^	−0.024	0.005	−0.876	−4.634	<0.01
		(Constants)	−20.837	2.698		−7.723	<0.01
Girls (*n* = 1910)	Nyingchi	BMI	1.143	0.320	1.308	3.568	<0.01
		BMI^2^	−0.027	0.007	−1.391	−3.795	<0.01
		(Constants)	−12.403	3.568		−3.476	<0.01
	Lhasa	BMI	1.528	0.349	1.540	4.373	<0.01
		BMI^2^	−0.035	0.008	−1.556	−4.419	<0.01
		(Constants)	−16.365	3.845		−4.256	<0.01
	Nagqu	BMI	0.523	0.303	0.547	1.728	0.085
		BMI^2^	−0.009	0.007	−0.405	−1.280	0.201
		(Constants)	−7.601	3.121		−2.436	0.015

## Data Availability

Due to the protection of subjects’ privacy, these data cannot be made public. The datasets generated during and/or analyzed during the current study are available from the corresponding author on reasonable request.
